# Predicting lung maturity in preterm rupture of membranes via lamellar bodies count from a vaginal pool: a cohort study

**DOI:** 10.1186/1477-7827-7-112

**Published:** 2009-10-14

**Authors:** Raed Salim, Noah Zafran, Zohar Nachum, Gali Garmi, Eliezer Shalev

**Affiliations:** 1 Department of Obstetrics and Gynecology, HaEmek Medical Center, Afula, Israel; 2 Rappaport Faculty of Medicine, Technion, Haifa, Israel

## Abstract

**Background:**

Amniocentesis is the accepted mode of attaining amniotic fluid to perform tests for fetal lung maturity. The purpose of this study was to validate a non-invasive fetal lung maturity test by counting lamellar bodies from a vaginal pool among women with preterm premature rupture of membranes.

**Methods:**

In a prospective study, amniotic fluid specimens were collected from a vaginal pool from women after preterm premature rupture of membranes with gestational age between 27 and 36 completed weeks. Receiver operating characteristics curve was estimated to assess the threshold of lamellar bodies' count that may predict fetal lung maturity.

**Results:**

Seventy-five specimens were collected of which 17 were between 32 to 34 weeks. A lamellar bodies' count of 28,000 or more predicted mature fetus 100% of the time (specificity) among all women and also among women between 32 to 34 weeks. The sensitivity was 72% among all and 92% when gestational age was between 32 to 34 weeks. A count of 8,000 or less, predicted respiratory distress syndrome with a sensitivity of 98% among the whole group.

**Conclusion:**

Counting of lamellar bodies in amniotic fluid from a vaginal pool may be used to predict fetal lung maturity.

## Background

Respiratory distress syndrome (RDS) is the most common complication suffered by preterm neonates [1]. Preterm premature rupture of membranes (P-PROM) is responsible for about a third of preterm deliveries [2]. Beside preterm delivery, P-PROM increases neonatal and maternal complications by increasing the risk of infection, cord accident and placental abruption [2]. According to the American College of Obstetricians and Gynecologists (ACOG), with P-PROM at 32-33 completed weeks of gestation, labor induction may be considered if fetal lung maturity is documented [2].

Amniocentesis is the accepted mode of attaining amniotic fluid to perform tests for fetal maturity. However, it is an invasive procedure with risks that include placental abruption, fetal maternal hemorrhage, infection and early onset of delivery [3]. The procedure is often technically challenging and potentially more complex when oligohydramnios is present as is often the case with P-PROM [1].

The purpose of this study was to test the validity of a non-invasive method for predicting fetal lung maturity among women with P-PROM. To do so, we performed a lamellar bodies (LB) count by drawing amniotic fluid from a vaginal pool and calculated a cutoff for LB concentration above which fetal lung maturity is likely. Among all fetal lung maturity tests, LB count was selected because the test can be performed with equipment found in most clinical analysis laboratories. Furthermore, such counting had been reported as reliably predicts fetal lung maturity, simple, rapid and inexpensive [4].

## Methods

A prospective study was held in the labor and delivery ward of the department of Obstetrics and Gynecology at Ha'Emek Medical Center in Afula, Israel, a university teaching hospital from January 2005 to January 2008. Pregnant women diagnosed to have P-PROM at a gestational age of 27 to 36 completed weeks were offered to participate in the study. Amniotic fluid was collected with a sterile speculum inserted into the posterior fornix. Only one specimen per subject was included. When twin gestations were included, the sample was drawn only from the presenting twin diagnosed with ruptured membranes and only the sampled twin was considered for analysis. Specimens, usually 0.5 cc or more, were transferred directly to test tubes and were processed within an hour of collection through a platelet channel of the cellular counter ADVIA 2120 (Bayer HealthCare, Tarrytown, NY, USA). Visual examination by a laboratory technician identified only clear specimens and therefore they were non-centrifuged. The test tube was placed on a stand in the cell counter and an automated aspiration of 157 μL of fluid was held. The test sample was separated into 5 different channels: 1) a channel for hemoglobin quantification, 2) a channel for quantifying red blood cells and platelets, 3) a basophil channel, 4) a peroxidase channel which gives the total white blood cell count and their differential count and 5) a reticulocyte channel. A platelets count was achieved by using a laser diode as a light source after spherization and fixation. The use of reagents and calibration of the cell counter were done according to the manufacturer's instruction.

Samples were excluded from analysis when an inadequate sample volume of amniotic fluid was collected or when the samples were otherwise unsatisfactory (ie, contained obvious mucus, grossly bloody or with hematocrit that exceeded 1%). Samples stained with meconium were also excluded from analysis because its presence usually provides a compelling reason for prompt delivery, irrespective of lung maturity status.

Other exclusion criteria included delivery more than two days after fluid analysis, maternal or fetal conditions that warrant expedite delivery before the sample collection, clinical amnionitis (P-PROM accompanied with fever, uterine tendeness and leukocytosis) and cases with inaccurate gestational age. Women who received betamethasone after the fluid analysis were also excluded.

The charts of mother-infant pairs were reviewed for demographic factors, diabetic status, betamethasone administration, and the presence of neonatal pulmonary and non-pulmonary morbidities. Gestational age was confirmed by either documented first trimester ultrasound or by known regular last menstrual period and a documented ultrasound in the second trimester.

The presence of RDS was defined clinically when the newborn developed tachypnea and grunting, had a chest radiography showing a diffuse reticular pattern and air bronchogram, and required oxygen for 24 hours or more. Neonates classified as having transient tachypnea of the newborn were not considered to have RDS. To investigate whether the frequency of non-pulmonary morbidity is related to the presence of lung maturity, we recorded all other cases of non-pulmonary morbidity. These included neonatal sepsis, necrotizing enterocolitis, intraventricular hemorrhage, neonatal hypoglycemia, anemia and thrombocytopenia. The time from delivery to discharge was also recorded. Obstetricians, neonatologists and radiologists were blinded to the results of the LB count attained from the exam.

The study was approved by the local institutional review board and the Israeli ministry of health research committee. Only women who agreed to sign an informed consent form were included in the analysis.

### Statistical analysis

Statistical analysis was based on logistic regression. A receiver operating characteristics (ROC) curve was estimated to assess the threshold of LB count that may predict RDS. The log transformation on LB was applied in the analyses where LB was used as a predictor. Stepwise logistic regressions were applied with RDS and with non-pulmonary morbidity as dependent variables to examine their relationships with other explanatory variables. Exact logistic regression was used for non-pulmonary morbidities. A *P *value < 0.05 was considered significant. The SAS software was used for the analyses.

## Results

During the study period there were 12,653 deliveries at our institution, with an 8.2% rate of preterm deliveries. Among all preterm deliveries, 282 (2.2%) were due to P-PROM between 27 to 36 completed weeks. Unsatisfactory samples, inadequate sample volume, cases where betamethasone was administered after the fluid analysis, delivery more than two days after fluid analysis, cases with maternal or fetal conditions that warranted expedite delivery, clinical amnionitis, inaccurate gestational age and cases in which women declined to participate were excluded. The remaining 75 mother-infant pairs were included in the analysis and made up our study cohort.

Maternal demographic and obstetric characteristics are presented in table 1. Twenty-seven women (36%) delivered before 34 weeks of gestation. Of these women, 9(33%) received a complete course of betamethasone (2 doses of 12 mg, 24 hours apart) within a week prior to delivery, seven (26%) received a complete course of bethametasone more than a week prior to delivery, nine (33%) received a partial course of bethametasone (1 dose of 12 mg) and two (8%) delivered shortly after admission and did not receive betamethasone.

**Table 1:** Demographic and obstetric characteristics of the 75 women included in the study.

Mean maternal age, years (SD)	30.5 (6.3)
Mean parity (SD)	2 (1.7)
Singleton (%)	57 (76)
Twins gestation (%)	18 (24)
Number of women with gestational diabetes (%)	6 (8)
Mean gestational age at rupture of membranes, weeks (SD)	33.9 (2.5)
Mean gestational age at delivery, weeks (SD)	34 (2.5)
Presentation at delivery	
Vertex (%)	68 (91)
Non-vertex (%)	7 (9)
Mode of delivery	
Vaginal (%)	65 (87)
Cesarean (%)	10 (13)

Neonatal outcomes are presented in table 2. Of the 13 neonates who developed RDS, four (30%) received surfactant. The mean number days of ventilation was 1.2 ± 4.1 days. Sixteen (21% of all neonates) received treatment with caffeine due to episodes of apnea. None of the neonates received nitric oxide. No correlation was found between the white blood cell count in the amniotic fluid and the presence of neonatal sepsis.

**Table 2:** Neonatal outcomes

Mean birth weight, grams (SD)	2172 (547)
Neonatal gender	
Males (%)	44 (59)
Females (%)	31 (41)
Number of neonates who developed respiratory distress syndrome (%)	13 (17)
Number of neonates who developed apnea (%)	13 (17)
Number of neonates who developed necrotizing enterocolitis (%)	4 (5)
Number of neonates who developed sepsis (%)	4 (5)
Number of neonates who developed intraventricular hemorrhage (%)	6 (8)
Number of neonates who developed thrombocytopenia (%)	2 (3)
Number of neonates who developed anemia (%)	11 (15)
Number of neonates who developed hypoglycemia (%)	4 (5)
Number of neonates who developed jaundice and treated with phototherapy (%)	57 (76)
Mean number of days from delivery to discharge (SD)	18.8 (18.1)
Number of neonatal deaths	0

**Figure 1:**
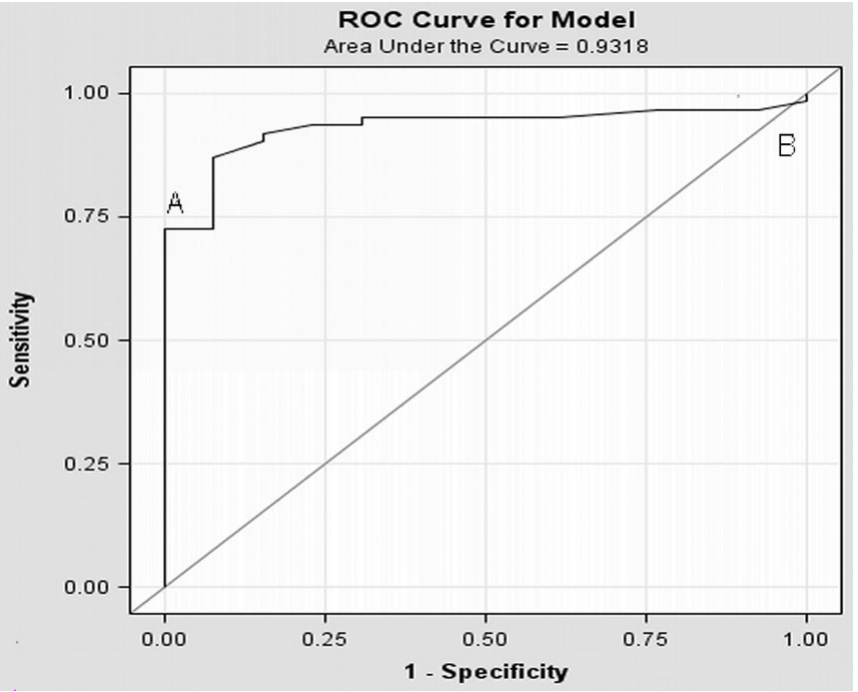
**ROC curve for lamellar bodies count with different cutoffs for different specificity and sensitivity**. (A) Lamellar bodies count at 28,000 (sensitivity 72% specificity 100%). (B) Lamellar bodies count of 8,000 (sensitivity of 98%).

The LB count ranged from 6,000 to 187,000 per microliter of amniotic fluid. When only the LB count was examined as a predictor of RDS, it showed that a count of 28,000 or more predicted fetal maturity 100% of the time (specificity) with a sensitivity of 72% (table 3). A count of 8,000 or less predicted RDS with a sensitivity of 98%. The AUC (area under the ROC) was 0.932 (figure 1). Among all women included, 17 presented with PROM between 32 to 34 weeks of gestation. Five neonates (29%) developed RDS. When the results were further sub-analyzed to this subgroup of women, a LB count of 28,000 or more predicted fetal lung maturity 100% of the time (specificity) with a sensitivity of 92% (table 4). The AUC was 0.967.

**Table 3:** Performance of the lamellar bodies count to predict the absence of clinical respiratory distress syndrome among all women

	**Sensitivity**	**Specificity**	PPV*	**NPV†**
LBC ≥ 28,000	72%	100%	100%	43%
LBC ≤ 8,000	98%	0%	82%	0%

*PPV = positive predictive value

†NPV = negative predictive value

**Table 4:** Performance of the lamellar bodies count to predict the absence of clinical respiratory distress syndrome among women between 32 to 34 weeks of gestation

	**Sensitivity**	**Specificity**	PPV*	**NPV†**
LBC ≥ 28000	91.7%	100%	100%	16.7%
LBC ≤ 16000	100%	60%	86%	0%

*PPV = positive predictive value

†NPV = negative predictive value

Logistic stepwise regression analysis excluding LB count showed that the best predictors of lung maturity were advanced gestational age (p = 0.02) and whether neonates were delivered vaginally or abdominally at the same gestational age (p = 0.03). Neonatal weight was found to be an additional significant explanatory variable in the model (P = 0.009) where log (LB) was used as a predictor for RDS (P = 0.0003). When comparing a model of predicting RDS with gestational age and mode of delivery with a model using LB count, the latter model had a superior AUC (0.9318 vs. 0.7940). There was no difference in the AUC when neonatal weight was added to LB count in the model (0.9318 vs. 0.9442).

The association between RDS and all the non-pulmonary morbidities was estimated by applying exact logistic regression with non-pulmonary morbidities as the dependent variable and RDS as the explanatory variable. A borderline significant positive association was found (P = 0.056). When applying logistic stepwise regression analysis, the only variable found to be significant in predicting the probability of non-pulmonary morbidities was the gestational age at delivery (P < 0.001).

## Discussion

Counting LB in amniotic fluid from a vaginal pool may be used for assessing fetal lung maturity among pregnant women admitted with P-PROM. A cutoff level of 28,000 predicted fetal lung maturity in 100% of the time with a sensitivity of 72%. A count of 8,000 or less predicted RDS with a sensitivity of 98%. As expected the incidence of RDS decreased significantly with advancing gestational age and was less common in neonates delivered vaginally compared with those delivered abdominally at the same gestational age. However, LB count was a stronger predictor of RDS than both gestational age and mode of delivery.

We also found a trend toward a reduced incidence of non-pulmonary morbidities among mature compared to immature neonates. This issue has been discussed previously in the literature, however, contradicting results were reported [5,6]. The main variable found to be significant in predicting the probability of non-pulmonary morbidities was the gestational age at delivery.

Different cutoff values for the LB count that predicts fetal lung maturity or RDS have been established and reported in the literature. Cutoffs that were established in this study are in concordance with other reports [7,8]. Discordances between cutoffs reported in the literature were largely due to differences in equipment and technique used and to whether the samples were centrifuged or not. Furthermore, failure to adhere strictly to a centrifugation protocol has also been reported as a reason that led to error [9]. In this study the samples were non-centrifuged. It has been reported that in the absence of obvious mucus or heavy meconium staining, processing non-centrifuged amniotic fluid specimens did not affect instrumentation adversely and did not affect the test performance [10,11]. Omission of this step saved time, further simplified the assay, and made interpretation of results uniform [9].

The decision to deliver women with P-PROM is based on the gestational age and fetal status. At 32 to 33 completed weeks of gestation, the risk of severe complications of prematurity is low if fetal pulmonary maturity is proven [12]. Beyond 34 weeks of gestation delivery is warranted according to the ACOG [2]. Still, there is a significant variation in clinical practice among different institutions in the management of women who present with P-PROM after 34 weeks [13,14]. According to the present study, a cutoff level of 28,000 of LB analyzed from a vaginal pool predicted fetal maturity in 100% of the time. The same cutoff was also valid when the results were further sub-analyzed to a subgroup of women with P-PROM between 32 to 34 weeks. Accordingly, institutions that practice expectant management at 34 weeks of gestation or more and induce labor at a greater gestational age may also benefit from the performance of this non-invasive test.

Amniocentesis is the accepted mode of attaining amniotic fluid for testing fetal lung maturity. However, it is an invasive procedure with risks and is often technically more complex when amniotic fluid volume is reduced, not to mention with oligohydramnios that may accompany P-PROM [1,3]. Obtaining amniotic fluid vaginally is non-invasive and fairly easy and can be done at the time of speculum examination.

The validity of amniotic fluid collected vaginally to predict fetal lung maturity has been reported previously [1,15,16]. Studies evaluated the performance of the TDx/TDxFLx fetal lung maturity II assay on amniotic fluid specimens collected vaginally from women with P-PROM showed that mature results predicted the absence of RDS with a high degree of accuracy [1,15]. Roiz-Hernandez et al evaluated the performance of a LB count from amniotic fluid specimens collected vaginally from a healthy pregnant population for predicting lung maturity. They concluded that counting LB is a quick, readily available, and very effective test. However, their population of pregnant women presented in labor without any associated medical condition or indication for assessing fetal lung maturity [16]. Compared to the latter group of women, we chose in this study to evaluate the performance of the LB count from amniotic fluid specimens collected vaginally among women with P-PROM. Of all fetal lung maturity tests we selected the LB count, because we concur with other reports that the test is fast, less technique dependent, less expensive, objective and can be performed easily in any laboratory with an electronic cell counter. Moreover, LB count performed as well as the other tests in predicting fetal lung maturity [4,17,18].

## Conclusion

Counting amniotic fluid LB from a vaginal pool is simple, quick, accessible and is readily useful in determining fetal lung maturity among pregnant women with P-PROM.

## Competing interests

The authors declare that they have no competing interests.

## Authors' contributions

RS and NZ conceived and designed the study, supervised the data collection, assisted in the analysis and drafted the manuscript; ZN and GG assisted in collection and maintenance of the data; ES assisted in conceiving, designing and analysis, and edited the manuscript. All authors read and approved the final manuscript.
